# Applying Least Absolute Shrinkage Selection Operator and Akaike Information Criterion Analysis to Find the Best Multiple Linear Regression Models between Climate Indices and Components of Cow’s Milk

**DOI:** 10.3390/foods5030052

**Published:** 2016-07-23

**Authors:** Mohammad Reza Marami Milani, Andreas Hense, Elham Rahmani, Angelika Ploeger

**Affiliations:** 1 Department of Organic Food Quality and Food Culture, University of Kassel, Nordbahnhofstr. 1a, 37213 Witzenhausen, Germany; a.ploeger@uni-kassel.de; 2 Meteorological Institute, University of Bonn, Auf dem Hügel 20, 53121 Bonn, Germany; ahense@uni-bonn.de (A.H.); erahmani@uni-bonn.de (E.R.)

**Keywords:** AIC, LASSO, climate indices, ESI, ETI, HLI, RRP, THI, linear regression model, milk components

## Abstract

This study focuses on multiple linear regression models relating six climate indices (temperature humidity THI, environmental stress ESI, equivalent temperature index ETI, heat load HLI, modified HLI (HLI _new_), and respiratory rate predictor RRP) with three main components of cow’s milk (yield, fat, and protein) for cows in Iran. The least absolute shrinkage selection operator (LASSO) and the Akaike information criterion (AIC) techniques are applied to select the best model for milk predictands with the smallest number of climate predictors. Uncertainty estimation is employed by applying bootstrapping through resampling. Cross validation is used to avoid over-fitting. Climatic parameters are calculated from the NASA-MERRA global atmospheric reanalysis. Milk data for the months from April to September, 2002 to 2010 are used. The best linear regression models are found in spring between milk yield as the predictand and THI, ESI, ETI, HLI, and RRP as predictors with *p*-value < 0.001 and *R*^2^ (0.50, 0.49) respectively. In summer, milk yield with independent variables of THI, ETI, and ESI show the highest relation (*p*-value < 0.001) with *R*^2^ (0.69). For fat and protein the results are only marginal. This method is suggested for the impact studies of climate variability/change on agriculture and food science fields when short-time series or data with large uncertainty are available.

## 1. Introduction

This study is about the linear relation between some climate parameters and cow’s milk compounds applied to data of milk production in Iran. Hoping to evaluate the environmental effects on animal husbandry and milk production, it would be better to consider as many environmental and physiological as well as milk compound variables as possible. In fact, animal husbandry and physiological performance is generally a result of combinations of variables affected by genetics, feed, housing/behavior as well as environmental conditions, which are very complex and potentially vary with time.

In general, because animal organisms evolve together with the environmental parameters in which they live, their physiological metabolism changes over time. For instance, Holstein cows are spread throughout the whole world, but in tropical regions they have been bred with different temperature tolerance levels compared to those Holsteins bred in regions with different climate conditions [1].

This shows that to study the interactions between environmental conditions and cattle husbandry and milk production as well as milk compounds is a complicated task and needs comprehensive information. Previous studies over the last two decades have mostly focused on climatic parameters and indices related to temperature and humidity, as more commonly used parameters, and the temperature humidity index (THI) as the most widespread indicator of heat stress [2,3]. Because of the indicated complexity due to animal adaptation ability and genetic variations, more predictors are required such as solar radiation in combination with wind speed, humidity, and temperature, and their combinations as indicators with equivalent temperature index (ETI), environmental stress index (ESI), heat load index (HLI), modified HLI (HLI _new_), and respiratory rate predictor index (RRP) [4]. Lacking a detailed physiological animal model to simulate the biophysical and biochemical influences of environmental conditions on the animal, the analysis has to rely on a statistical approach. Unfortunately, the available data sample sizes do not allow the use of a wide range of environmental predictors in a classical regression approach. Therefore, the physiologically motivated indicators are combined with the recently developed method of least absolute shrinkage and selection operator (LASSO). It is a purely data driven predictor selection method. It does not share the ambiguities of other predictor selection methods like stepwise regression, which, e.g., depend on the ordering of the predictors [5].

According to this motivation and the requirement for new, better and more indices, in this study, some more climatic indices are calculated from several climate parameters to have more combinations of the parameters, which might have an effect on the quantity of milk compounds. THI, ETI, ESI, HLI, HLI _new_ and RRP indices are used in this research to consider the influences of temperature, humidity, wind speed, and solar radiation on components in cow´s milk. These indices are considered as the independent variables or predictors in a set of multiple linear regression equations for milk components. The most suitable merged statistical model between all indices as predictors and milk parameters as predictands are obtained through multiple linear regression models under the LASSO constraint.

Moran and Epstein evaluated ESI as the best suitable heat stress index for hot climate in both dry and wet conditions [6]. The HLI was suggested for the first time by Gaughan et al. in 2002. Since 2008, HLI has been modified (HLI _new_) by Gaughan et al. [7,8,9]. Silva et al. also suggested HLI for the tropical regions [8]. We decided to study both indices (HLI and HLI _new_) to compare them with each other and with other indices. Marami et al. [2,4] report on correlations between different climate indices (THI, ETI, ESI, HLI, HLI _new,_ and RRP) and milk compounds (fat, protein, and milk yield) using the bootstrap technique. They found that the use of a single index is valid only under certain and specific climate conditions. The results in Table 1 and Table 2 summarize that each index alone does show very different correlations with milk compounds.

The main question to be answered is to find more accurate predictions linking several climatic indices and parameters, which characterize the quality of milk. The use of single indices has been proven to show realistic and interpretable results [2,4]. Here, we will test if the combination of certain climate indices can provide a better statistical model than those reported in Table 1 and Table 2. This will analyze the information content of the set of climate indices with respect to the milk compounds beyond the simple co-linearity of the single indices.

Finding effective predictors can lead to an accurate model with the aim of studying the impact of climate change on milk components to predict for the near future.

Generally, our research follows some main aims: (1) Introducing specific methods for data preparation and statistical analysis to cope with uncertainties in time series data sets with statistical problems such as data gaps, expensive or difficult data collection procedures, and the small quantity of samples and (2) selecting the best model with the smallest number of predictors by applying recent statistical techniques and avoiding misinterpretations due to overfitting to achieve more suitable combinations of climate indices with milk compounds (milk yield, and protein) in spring and summer in terms of a better statistical model measured by higher correlation *R*^2^ or smaller *p*-value.

Hypothesis of this study consisted of opening avenues for potential applications, e.g., designing dairy factories according to the economic justifications and feeding system to be managed with less risk of climate variability, especially in developing countries.

To meet the requirements for hypothesis No. (2), LASSO [5] and AIC statistics techniques are applied and cross validated to avoid errors by overfitting. To consider uncertainty and constructing confidence intervals, bootstrap method is applied which is a field of active research in statistics, particularly for dependent data [10,11].

The climate data used in this study included daily averages of solar radiation (SR), two meter height temperature (T_2m_), dew point (T_d_), relative humidity (RH), wind speed (V), sea level pressure (p), and specific humidity (q) which are taken from the NASA-Modern Era Retrospective-Analysis for Research and Applications (NASA-MERRA) reanalysis [12].

## 2. The Region of Study and Data Basis

The geographic region of this study is Iran, a country with variety in topography and different climate conditions. The Caspian Sea in the north, Persian Gulf and Gulf of Oman in the south, Alborz and Zagros Mountains in the north and northwest-southeast, and the two famous deserts Dasht-e-Kavir in the central plateau and Dasht-e-Lut in the southeast are the main geographical resources in Iran.

Two main data bases, climate and milk data, are taken for this study. Climate data are taken from the MERRA reanalysis dataset. MERRA provides gridded data with a resolution of 1/2 degrees latitude and 2/3 degrees longitude covering all meteorological quantities in a physical and statistical self-consistent manner. This is achieved by merging observations from satellite and meteorological stations using a meteorological forecast model [12]. This means that the meteorological variables such as temperature, wind, and humidity obey the physical relationships among each other, which are dictated by the physical laws of fluid dynamics. Additionally, the meteorological variables are combined into three-dimensional spatial temporal patterns in between the observed points, which share comparable statistics, like correlations, among the space and time points as the observations.

Climate data for the same months and years as milk data have been extracted. The four new climate indices Environment Stress Index (ESI), Heat Load Index (HLI), modified Heat Load Index (HLI _new_), and Respiratory Rate Predictor (RRP) are calculated from the corresponding climate data parameters based on the daily meteorological grid point data.

Regarding milk data, industrial herd stations in Iran are investigated. In total 18,295 industrial herd stations are active out of 25,353 herd stations with a capacity of about 1.3 million cows. 66% of the cows in Iran are pure Holstein, Jersey and Brown Swiss, 27% are hybrids and 7% are home born cows. Corn, grain, alfalfa, wheat chaff, silage, wheat, soybean, cottonseed, and other forages are important ingredients in the cows’ feed in Iran [13].

### 2.1. Milk Data

Milk data is preprocessed considering some important terms such as genetics, cow age, environmental conditions, season, lactation, pregnancy, and feeding management from the industrial herd stations with good health services and under controlled condition and veterinary care.

In this study, the monthly average of test-day (TD) records of milk yield (kg) are used; TD expresses the day that the cow was milked. Additionally, three times weekly records of fat and protein content (g/100 mL milk) had been collected between 2002 and 2010 from almost 600 industrial herd stations of Holstein cows, with herd sizes varying between 75 to 200 cows, with access to grazing during April to September as a base feeding. The final monthly averaged milk data are in a matrix of 3 × 936,227 individuals for milk yield, fat, and protein. Data gathering was under the conditions that cows were on days 4 to 305 milking time and between three and six calves in their lifetime without mastitis problem during the whole period of study. Records, which indicate that a cow was in a dry period or had mastitis, were omitted from the primary row data set [2,4].

### 2.2. Climate Indices

The equations used to compute the climate indices are presented in Table 3. The necessary input data are taken from the MERRA data set on all grid points covering the study area. The equations are the basis to investigate their relationship in a multiple linear regression model with milk compounds. The indices used are the temperature humidity index (THI), the equivalent temperature index (ETI), the environmental stress index (ESI), the heat load index (HLI), the modified HLI (HLI _new_), and the respiratory rate predictor index (RRP). Table 4 lists the necessary input variables taken from the MERRA database of the indices presented in Table 3.

## 3. Materials and Methods

**Figure 1:**
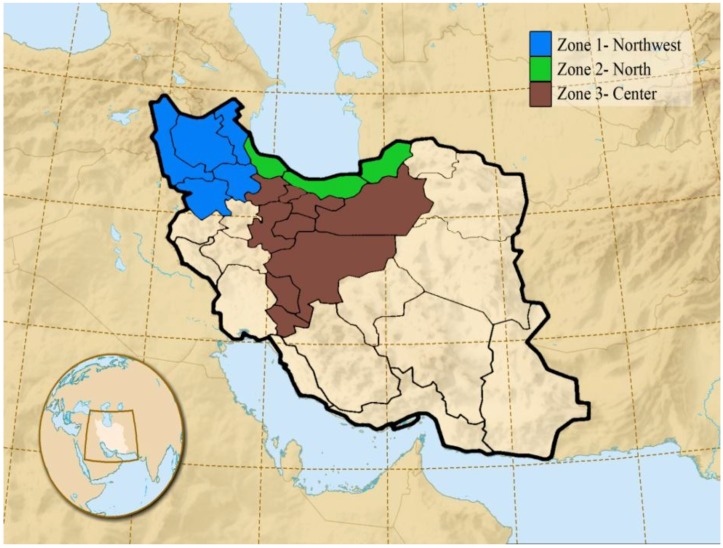
Classification of three study zones according to climate conditions in Iran.

In regards to climate variability in Iran, three different climatic zones in the northwest, north, and center of Iran are selected with diverse climate conditions including cold semi-arid, Caspian mild and humid, semi warm, and semi-arid. The data of climatic and milk parameters are selected in each zone separately during 2002 to 2010 for summer and spring. Figure 1 shows the selected climatic zones in the study domain.

**Figure 2:**
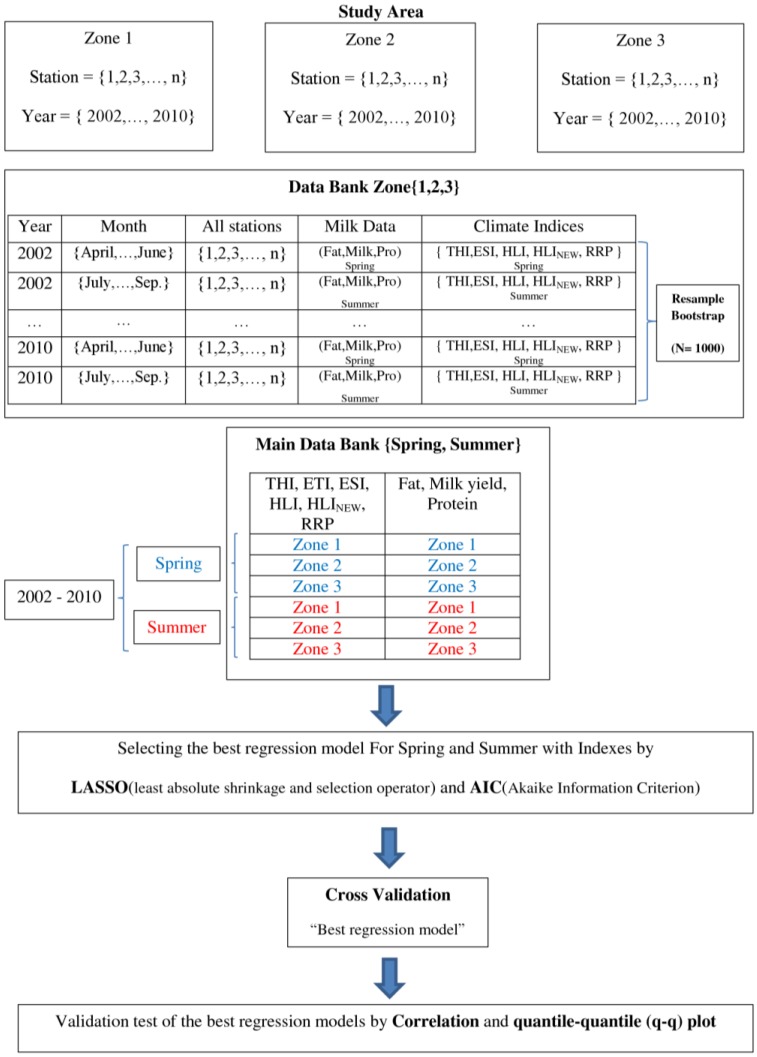
The flowchart of the data set of climatic and milk parameters and methodology.

To consider uncertainty, non-parametric bootstrap technique is applied to the monthly mean data of milk yield, fat, and protein concentration during 2002 and 2010 in each zone separately by generating 1000 samples which is presented in Figure 2.

For both the correlation analysis and the multiple linear regression, the data are normalized to a mean of zero and a standard deviation of one.

In this work, we model the observed milk components employing methods which take different models and parameters into account.

To measure the “distance” between a specific model based on the meteorological data and the observed milk variables, the “Kullback-Leibler Information” (K-L distance) together with the Akaike’s Information Criterion (AIC) are used. The AIC identifies the model that minimizes K-L distance.

In order to select the best model with the smallest number of predictors, the LASSO and the AIC are combined. To avoid overfitting and to test the validity of the obtained model, cross validation is applied on the selected model. For presenting graphically the results in an appropriate statistical sense, quantile-quantile plots are used.

All the analyses in this study are implemented using R version 3.2.2, which provides various packages for statistical data analysis, calculation and graphical display [19]. THI, ETI, ESI, HLI, HLI _new,_ and RRP are considered as independent variables (predictors) in multiple linear regression equations, and milk components (milk yield, fat, and protein) are considered as dependent variables (predictands).

### 3.1. Least Absolute Shrinkage and Selection Operator Technique (LASSO)

Tibshirani [5] proposed a new method for estimation and predictor selection in linear models. The LASSO is an effective technique for shrinkage and selection method for linear regression. It minimizes the residual sum of squares like in classical linear regression to determine the unknown regression coefficients related to each predictor. As an extension to classical regression, the regression coefficients are constrained by the sum of the modulus or absolute values of the coefficients being as small as possible. The advantages of this combination of techniques is to reduce the regression coefficients as much as possible (shrinkage) and, depending on the data, setting some regression coefficients exactly to zero (selection), which is specifically achieved by the sum of the modulus of the regression coefficients.

In this study the LASSO method is used for finding the best regression models between the main compounds of milk (fat, protein, and milk yield) as predictands and environmental indices as predictors. To that aim, we applied an available package of glmnet in R [20].

LASSO presents the regression coefficients $\beta_{k}$ (Equation (1)) as a function of a regularization parameter Lambda (λ), which determines the influence of the classical least squares contribution (first sum over n in Equation (2)) relative to the sum of modulus of the coefficients (second sum over k in Equation (2)).

(1) $$Y\sim \sum_{k}\beta_{k}\;X_{k}+e$$

(2) $$J\left(\beta_{k}\right)=\frac{1}{N}\;\overset{N}{\underset{n=1}{\sum }}\;{\left(Y_{n}-\sum_{k}\beta_{k}\;X_{k,n}\right)}^{2}+\lambda \;\sum_{k}\left|\beta_{k}\right|$$

Minimization of the function $J\left(\beta_{k}\right)$ for varying λ values will select non zero $\beta_{k}$ solely on the basis of the data $\left(Y_{n},\;X_{k,n}\right)$.

By increasing λ from zero (LASSO switched off or classical solution) to higher values (putting more weight on the absolute value constraint), glmnet sets increasingly more coefficients to zero, thereby removing them from the model. If for a range of λ values a constant number k of independent predictor variables is selected, we consider this potentially the best multiple linear regression model.

### 3.2. Akaike Information Criterion (AIC)

The Akaike Information Criterion (AIC) is a method for selecting a model from a set of models based on bias minimizing through Kullback-Leibler distance. Kullback and Leibler [21] measured a divergence which defines the distance measure between two probability distributions over the same event space. AIC value provides a mean which estimates the quality of each model in comparison to the other models. It selects the model with largest likelihood under the constraint of the smallest number of predictors. It was presented by Akaike in 1973 and is defined as:

(3) AIC=2k−2ln(L)

*k* is the number of parameters in the model and *L* is the likelihood which deals with the goodness of fit. AIC is negatively oriented with smaller AIC values representing better models [22,23]. From the potentially best multiple linear regression models selected by the LASSO algorithm, the best model is defined as the one with the lowest AIC.

### 3.3. Cross Validating the Best Regression Model

**Figure 3:**
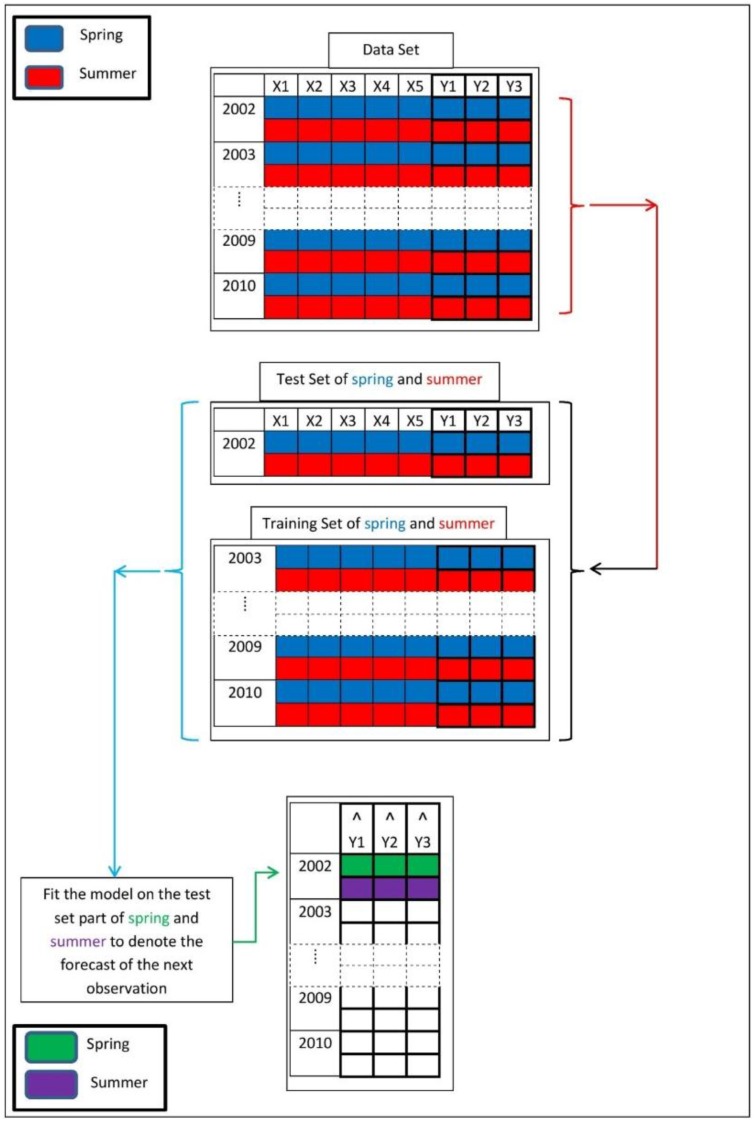
Cross validation procedure.

Cross validation is a model evaluation technique which prevents overfitting by applying the model to the data that are not involved in the fitting [24,25]. In cross validation, we withhold one year (testing set) of data and estimate the regression model with the rest of data (training set) according to the rules described in the previous subsections. After selecting the best regression model by the LASSO and AIC, the withheld predictors are used to compute the milk component variables which can be compared to the withheld observations. This is done for all data sets from 2002 to 2010 for spring and summer separately. Figure 3 presents the cross validation process in this study. Afterward, the accuracy of the predicted model versus the observation is accomplished via Pearson correlation and quantile-quantile (QQ) plot [26,27].

## 4. Results

In this section, the best models are presented through multiple linear regression analysis between climate indices and milk components in spring and summer, which are preselected through LASSO and finalized by AIC methods. Significant relationships in spring and summer could be identified between climate indices and milk yield through these methods.

The multiple linear regression models achieve higher correlation, *R*^2^ and smaller *p*-value for milk yield than the simple linear regressions presented in Table 1 and Table 2. For fat and protein, no significant model is suggested which performs better than what has been found with a single index by Marami et al. [2,4] in the previous studies. This is comparable to Knapp and Grummer [28] and Roman-Ponce et al. [29] who also did not find any significant relationship between fat variations and heat stress variables.

### 4.1. The Best Linear Regression Model between Climate Indices and Milk Components in Spring

**Figure 4:**
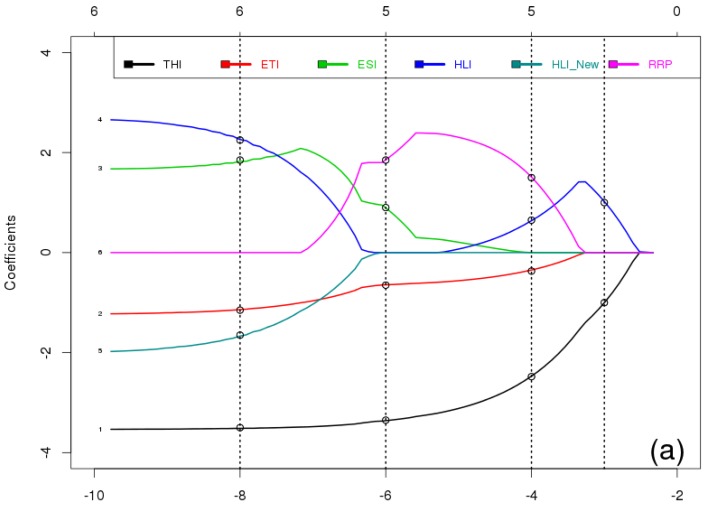

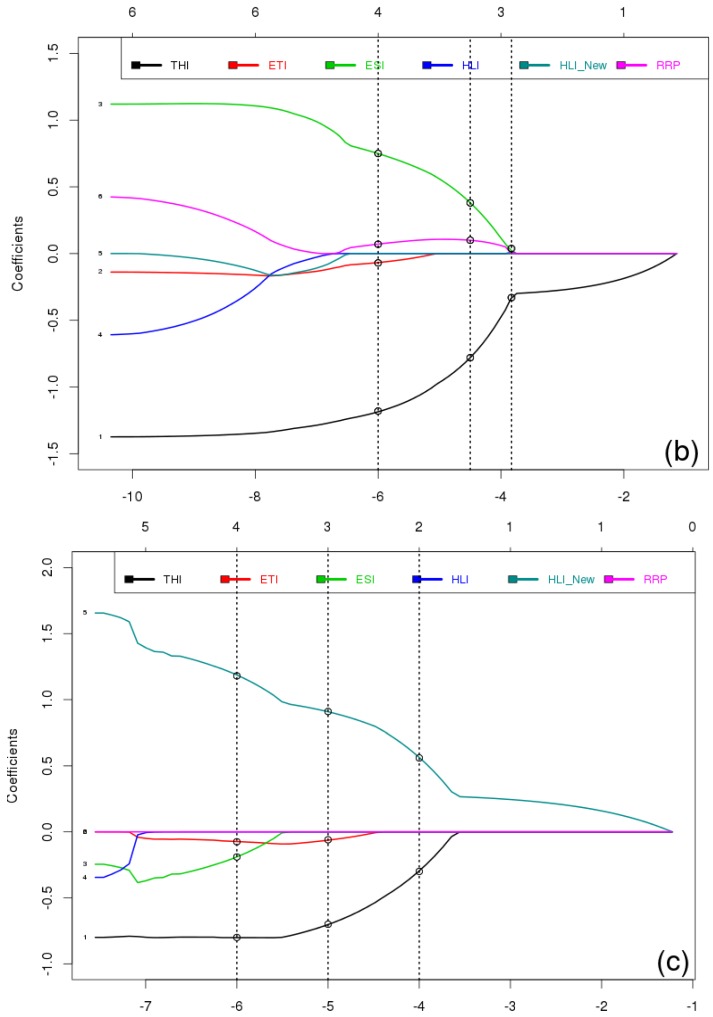
Least Absolute Shrinkage and Selection Operator (LASSO) analysis to find the best multiple linear regression model between climate indices and milk yield (**a**), fat (**b**), and protein (**c**), from 2002 to 2010 in spring. On the bottom *X* axis, the shrinkage regularization factor is given in logarithmic units (log λ). The number on top of the figure indicates the number of Lasso selected predictors at the log λ value.

Results of the analysis in spring are shown in Figure 4 and Table 5. Figure 4 shows the results of LASSO analysis between climate indices and milk yield (a), fat (b), and protein (c) from 2002 to 2010 in spring. On the bottom the shrinkage regularization factor is given in logarithmic units (log λ) with the smallest negative values indicating a very small weight of the LASSO regularization or an almost classical multiple linear regression with all predictors included. The axis on top of the figure indicates the numbers of LASSO selected predictors at the given log λ value.

As seen in Figure 4a, the highest weight of LASSO in the right side of the figure has selected two predictors for milk yield which are THI and HLI. Then, by moving to the left hand side of the figure, the weight of LASSO regularization decrease and selects four predictors (THI, ETI, HLI, and RRP), and then a different four predictors (THI, ETI, ESI, and RRP). With the least weight of LASSO, it selects five predictors for milk yield: THI, HLI _New_, ETI, ESI, and HLI. In the next step AIC will be used to select the best multiple model within the selected models of LASSO.

Table 5 presents values of AIC, *R*^2^ and *p*-values, and final selection of the best model by AIC within the preselected predictors by LASSO for the analysis of the spring data. The AIC values can be directly compared because all data (predictors as well as predictands) have been normalized to a mean of zero and a standard deviation of one.

(4) Milk yield Spring(Mod3)=−3.58 THI−0.77 ETI+5.14 ESI−2.3 RRP+e

(5) Milk yield Spring(Mod2)=−3.485 THI−0.68 ETI−0.295 HLI+3.13 RRP+e

Standard error of the estimates is calculated through Equation (6). Y is observed value, Y′ is predicted value and N is the number of pairs of scores.

(6) $$\sigma_{est}=\sqrt{\frac{\sum {\left(Y-Y\prime \right)}^{2}}{N}}$$

Standard error of estimate for model Equations (4) and (5) are calculated at 0.706 and 0.705 respectively. In both of the models, maximum negative effect belongs to THI with milk yield. The inclusion of the additional predictors raises the correlation from 0.22 for THI vs. yield in spring (Table 1) to 0.7, proving the added value of the other climate indices.

### 4.2. The Best Linear Regression Model between Climate Indices and Milk Components in Summer

**Figure 5:**
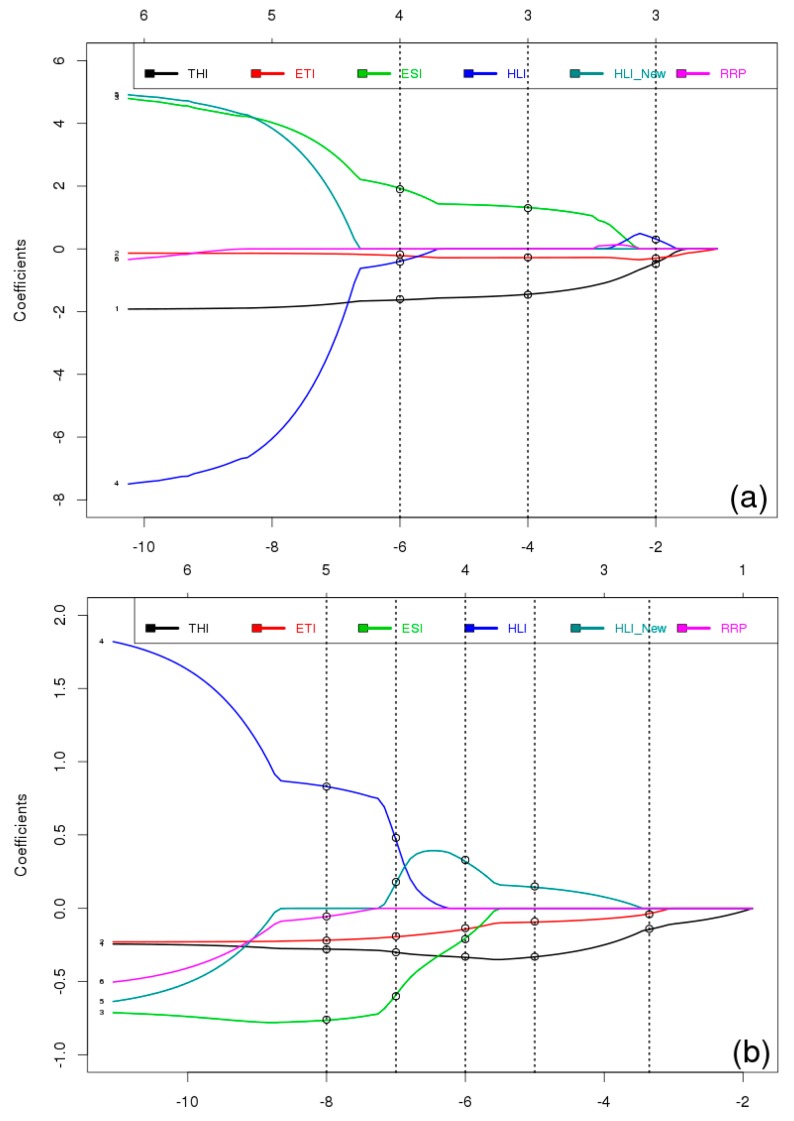

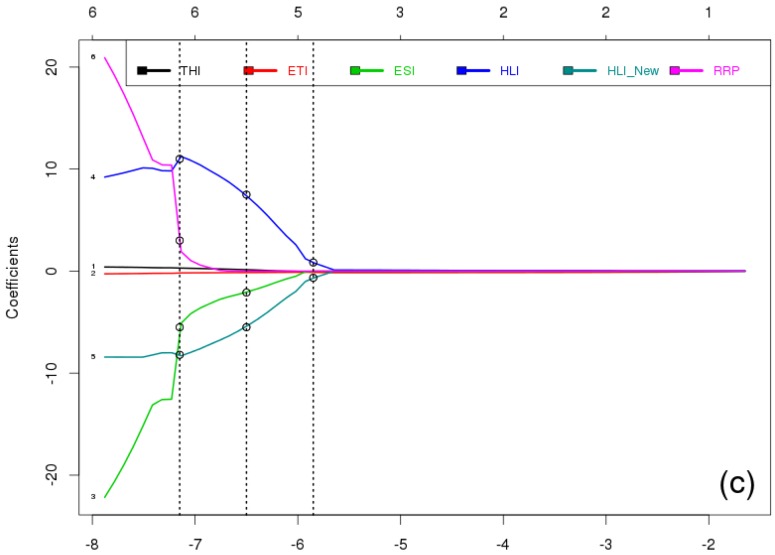
LASSO analysis to find the best linear model between climate indices and milk yield (**a**), fat (**b**), and protein (**c**) from 2002 to 2010 in summer. On the bottom X axis, the shrinkage regularization factor is given in logarithmic units (log λ). The number on top of the figure indicates the number of Lasso selected predictors at the log λ value.

**Figure 6:**
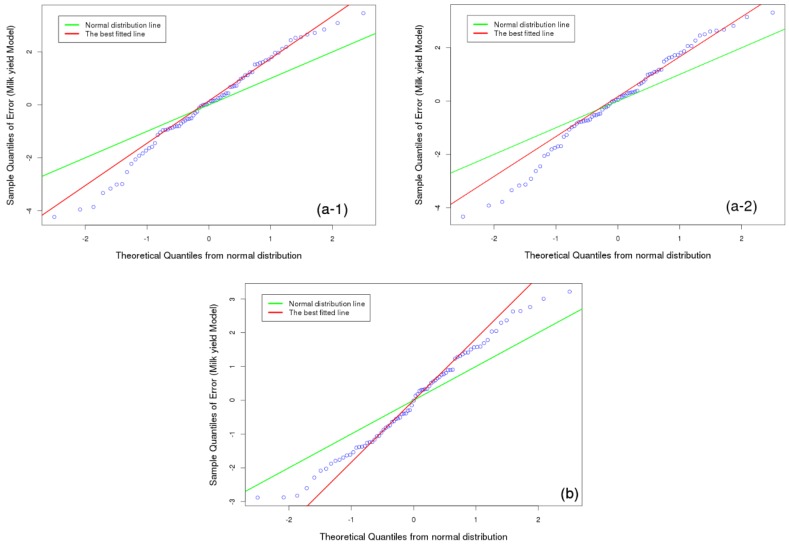
Quantile-Quantile (Q-Q) plot of the best models for milk yield in spring (**a**) and summer (**b**).

Similar to the spring analysis, results of the analysis for summer are shown in Figure 5 and Figure 6. Figure 5 shows the results of LASSO analysis between climate indices and milk yield (a), fat (b), and protein (c) from 2002 to 2010 in summer.

Referring to Figure 5a for milk yield, the highest weight of LASSO in the right side of the figure has selected three predictors for milk yield which are THI, ETI, and HLI. Then by moving to the left hand side of the figure, the weight of LASSO regularization decreases and selects another three predictors (THI, ETI, and ESI). With the least weight of LASSO, it selects four predictors (THI, ETI, ESI, and HLI). Then, in the next step, AIC will select the best multiple model within the selected models of LASSO.

Table 6 presents values of AIC, *R*^2^ and *p*-values, and final selection of the best model by AIC within the preselected predictors by LASSO in summer.

The best multiple linear regression models of milk yield in summer are presented as

(7) Milk yield Summer(Mod3)=−1.7 THI−0.126 ETI+2.58 ESI−0.91 HLI+e

(8) Milk yield Summer(Mod2)=−1.6 THI−0.28 ETI+1.47 ESI+e

In both models, like as in spring, the highest negative relationship existed between THI with milk yield. Standard error of estimate (Equation (6)) for both models in summer is calculated at 0.546.

In contrast to the results for spring, the models (7) and (8) have a different number of predictors. In this case, one would invoke Occam’s razor [30] to favor the model (8) with three predictors, all other selection criteria being equal.

### 4.3. Model Verification and Validation

The quality of the models are assessed by applying a quantile-quantile (Q-Q) plot, correlation coefficient, and mean squared error skill score (MSES) by Equation (9) to see how close a fitted line is to the data points. Here Y are the verifying observations, Y’ the predicted values, and $\bar{Y}$ the climatological mean as the reference prediction.

(9) $$\text{MSES}=1-\left[\frac{MSE\left(Y^{\prime },Y\right)}{MSE\left(Y,\bar{Y}\right)}\right]$$

Quantile-Quantile plots the sorted, observed quantiles of the residuals Y′-Y against the theoretically expected quantiles of a normal distribution with a mean of zero and a standard deviation of 1. If the points align along a linear increasing function, the requirement that the residuals are realizations of normally distributed random variables is well fulfilled. This checks, a-posteriori, the basic assumptions of the multiple linear regression analysis. The slope of the linear function corresponds to the standard deviation of the residuals Y′-Y.

Figure 6 (a-1, a-2) and (b) present the Q-Q plots of the residual errors Y-Y′ of the best models vs. the theoretical quantiles of a random normal variate with mean zero and variance 1 for milk yield in spring (Equations (4) and (5)) and summer (Equation (8)).

In both cases, the prerequisites of the multiple regression analysis of normally distributed residuals are well fulfilled. Such a model quality could not be achieved with a single predictor model by Marami et al. [4], nor could it be selected by prior methods.

The MSES should be equal to or less than *R*^2^ of the model. In this study MSES of milk yield in spring (a-1, a-2) and summer (b) are 0.49, 0.49 and 0.69 respectively.

**Figure 7:**
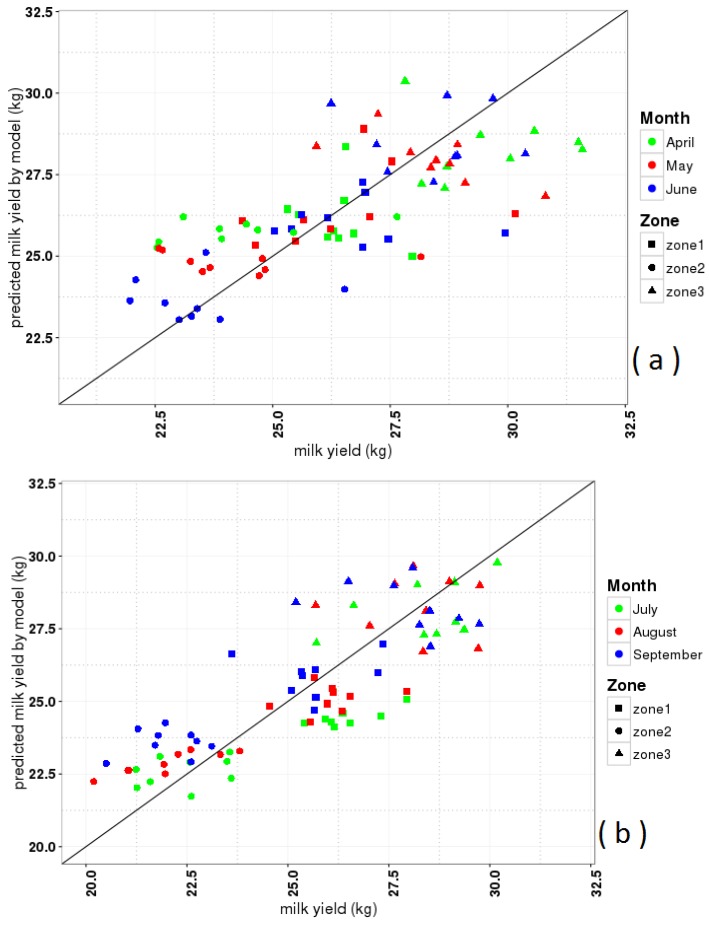
Layout data (observed) of milk yield vs. predicted milk yield data by models in spring (**a**) and summer (**b**). Months indicated by colors and zones (Figure 1) by different symbols.

Figure 7a,b show the outcome of the best models for milk yield in spring (Equations (4) and (5) and summer (Equation (8)). They visually represent the observed and predicted values l in dimensional units. In spring, the correlation between predicted milk yield from the models and observed data is 0.7 (*R*^2^ = 0.49, Table 5), with *p*-value < 0.001. For the summer, it increases to a higher value of 0.84 (*R*^2^ = 0.7, Table 6), with *p*-value < 0.001. The models of milk yield in spring and summer explains almost 50% and 70% of the linear dependency vs. 50% and 30% for unexplained scatter respectively. Such a model quality could not be achieved with a single predictor model by Marami et al. [4] nor could it be selected by prior methods.

This figure shows that the distribution of data in different climatic zones (Figure 1) and months (from 2002 to 2010) equally contributed to the overall correlation between observations and predicted data, indicating a robustness of the results.

## 5. Discussion and Conclusions

According to cow characteristic changes and adaptation abilities under different conditions, the effect of climate variability on animal products and quantities of nutrients and yield is very hard to work out in full detail [1].

The assumption is that the interactional effect of air temperature, humidity, wind speed, and solar radiation make a critical point of heat stress with a negative effect on cows’ body metabolism. It was reported in 1988 by Dupre et al. [31] that 11% of metabolic rate is altered by changing 1 °C in body temperature. We do not consider indirect effects of climate variability, e.g., variable nutrient contents of forage crops through different growing conditions.

Heat stress has a negative effect on hypohydration, endurance and thermoregulatory control systems, and changes the blood volume and plasma osmolality of cows. As well, it affects rumen function, hormonal status, and maintenance energy requirements; it increases respiratory rate and sweating, and reduces feed intake and activity. Cows under heat stress lose large quantities of potassium which could lead to an increase on blood acidity [32,33,34,35]. To moderate the effects of heat stress, cows might be protected from direct solar radiation using a combination of fans and wetting, improving the cooling system, and developing nutritional strategies [36]. Other heat stress management possibilities for cows are to stay in the barn or to graze on pasture in the pasturing system [37].

In this study, we suggested a statistical approach to find out the best multiple linear regression model for the influence of heat stress on key components of milk. Additionally, the aim was to consider uncertainties as much as possible.

Here, the effect of heat stress on the fundamental milk compounds milk yield, fat, and protein contents were investigated. We obtained three suitable models (Equations (4), (5) and (8)) as the best multiple models between climate indices and milk yield for spring and summer.

In total, six different predictors of heat stress indicators are introduced which summarize, as much as possible, the physiological knowledge of the reaction of cattle on environmental forcing. The appropriate mix of predictors is determined by a solely data driven approach: the least absolute shrinkage and the selection operator LASSO. The result of multiple linear regression models lead to correlation and *R*^2^ values which are highly promoted and provide more suitable and significant models than those suggested by single indices in Marami et al. [2,4] and other studies.

We found that the single use of indices is valid only under certain and specific climate conditions. The results exhibited that each index separately shows very different correlation with milk compounds although these indices have high correlation with each other (Marami et al. [2,4]). Coping with this problem, in 2002 Brake and Bates [38] developed a method of limiting metabolic rate which allows all heat stress indices to be compared with each other virtually.

In contrast with Brake and Bates, we eliminated this problem by applying multiple linear regression models to develop new indices through the combination of single heat stress indices and climate parameters together with considering uncertainties as much as possible.

For fat and protein, no significant model is better than what is suggested by Marami et al. [2,4] in the previous studies. For milk yield, a clearly superior model was identified. Note that the regression coefficients in the three models (Equations (4), (5) and (8)) can be directly compared due to the normalization of predictors as well as predictands. For both spring and summer, THI is the major driving negative component, meaning that an enhanced heat stress expressed by the temperature and humidity effects alone [31] leads to a reduction in milk yield. The difference between both seasons is that the THI is about two times more effective in yield reduction during spring than during summer. The second identified component with a negative influence upon milk yield is modeled through ETI. This index differs from THI by the inclusion of wind velocity which by the ventilation effect apparently contributes additional information beyond the co-linearity with THI. Further influences in variations of the milk yield are modeled with ESI, HLI, and RRP depending on the season, with ESI having always a positive effect upon milk yield. All three indices differ from the THI and ETI through their inclusion of solar radiation. This also gives an explanation as to why the combined indices provide a superior model in terms of explained variance of milk yield: It is the combination of temperature, humidity based heat stress with ventilation effects and the solar radiation as an additional heat source, which together serve as a driver of the metabolic processes listed above. The results of this statistical analysis can not completely rule out that the indirect effects, e.g., through nutrient content, play a role. However, the definition of the indices in use is with emphasis on the metabolic effects.

It is strongly suggested that investigating the effects of climate variability on fundamental milk components is done separately on every single parameter of milk. The critical points of heat stress indices need to be updated for different climate conditions because of characteristic changes and cows´ adaptation ability. It is very important because it is the starting point of the negative effects on milk compounds [1].

Based on the discussion above, the authors strongly recommend new indices considering different climate conditions with more predictors, such as sunshine duration, quality of cows’ feed, color of skin with attention to large black spots, and some categorical predictors such as breed, welfare facility, and management systems with categorical codes to control the critical heat stress conditions.

## Figures and Tables

**Table 1:** Correlation between climate indices (Temperature Humidity Index (THI), Equivalent Temperature Index (ETI), Environmental Stress Index (ESI), Heat Load Index (HLI), modified HLI (HLI _new_), and Respiratory Rate Predictor index (RRP)) and milk compounds (fat, milk yield, and protein) in spring (* is *p*-value < 0.05, ^§^ is *p*-value < 0.1 and ^#^ is not significant). Taken from [2,4].

	THI	ETI	ESI	HLI	HLI _new_	RRP
Fat	−0.47 *	0.42 *	−0.41 *	−0.41 *	−0.40 *	−0.41 *
Milk yield	−0.22 ^#^	−0.21 ^§^	0.26 *	0.27 ^§^	0.26 ^§^	0.26 ^§^
Protein	0.37 ^§^	−0.43 ^§^	0.41 *	0.42 *	0.44 *	0.41 *

**Table 2:** Correlation between climate indices (THI, ETI, ESI, HLI, HLI _new,_ and RRP) and milk compounds (fat, milk yield, and protein) in summer (* is *p*-value < 0.05, ^§^ is *p*-value < 0.1 and ^#^ is not significant). Taken from [2,4].

	THI	ETI	ESI	HLI	HLI _new_	RRP
Fat	−0.32 *	0.17 *	−0.23 *	−0.21 ^§^	−0.21 ^§^	−0.23 *
Milk yield	−0.26 ^#^	−0.43 ^§^	0.43 *	0.43 *	0.41 *	0.43 *
Protein	0.31 ^§^	−0.34 ^§^	0.37 *	0.36 ^§^	0.34 ^§^	0.37 *

**Table 3:** Definition equations of the climate indices (THI, ETI, ESI, HLI, HLI new, and RRP) used for investigating the influence of environmental influences upon milk components in this study.

Climate Index	Equation
Temperature Humidity Index [14,15,16]	$THI=41.5+T+0.36T_{d}$ *where* $T_{d}={\left(\frac{RH}{4}\right)}^{\frac{1}{8}}\times [112+(\frac{9T}{40})]+\frac{T}{40}-112$, $RH=e/e_{w}^{*}$ e=1.6077×p×q, $e_{w}^{*}=6.1078\exp\left[\left(17.1\times T)/(235+T\right)\right]$
Equivalent Temperature Index [1]	$\begin{array}{l} ETI=27.88-0.456T+0.010754T^{2}-0.4905RH+0.00088RH^{2}+\\ 1.1507V-0.12645V^{2}+0.019876T\times RH-0.046313T\times V \end{array}$
Environmental Stress Index [17]	$\begin{array}{l} ESI=0.63T_{a}-0.03RH+0.002SR+0.0054T_{a}\times RH-\\ 0.073{\left(0.1+SR\right)}^{-1} \end{array}$
Heat Load Index [7,8]	$HLI=33.2+0.2RH+1.2T_{g}^{*}-{\left(0.82V\right)}^{0.1}-\log(0.4V^{2}+0.0001)$ *where* $T_{g}^{*}=1.33T-2.65T^{0.5}+3.21\log(SR+1)+3.5$
Modified Heat Load Index [9]	$HLI_{new}=10.66+0.28RH+1.3T_{g}^{*}-V$ *when* $T_{g}^{*}$ < 25 (°C)
	$HLI_{new}=8.62+0.38RH+1.55T_{g}^{*}-0.5V+e^{2.4-V}$ *when* $T_{g}^{*}$ > 25 (°C)
Respiratory Rate Predictor Index [18]	RRP=5.4T+0.58RH−0.63V+0.024SR−110.9

**Table 4:** Naming of the MERRA input variables, their definitions, and units used in the computation of the climate indices (THI, ETI, ESI, HLI, HLI _new_ and RRP) in Table 3.

Parameter	Definition
T	2 m height temperature (°C)
T_d_	Dew point temperature (°C)
RH	Relative humidity (%)
e	Vapour pressure (hPa)
$e_{w}^{*}$	Saturation vapour pressure (hPa)
p	Sea level pressure (hPa)
q	specific humidity
v	wind speed (m/s)
T_a_	ambient temperature (°C)
SR	solar radiation (wm^−2^)

**Table 5:** Results of LASSO and Akaike Information Criterion (AIC) analysis for finding the best multiple regression model between milk parameters and climatic indices in spring. Mod1 to Mod4 in “Milk yield” refers to the dashed lines in Figure 4a; Mod1 to Mod3 in “Fat” refers to Figure 4b, and Mod1 to Mod3 in “Protein” refers to Figure 4c.

Layout Parameter	Preselected Predictors By LASSO	AIC	Δ AIC	Model Selected by AIC	*R*^2^	*p*-Value
Milk yield	Mod 1: THI , HLI Mod 2: THI, ETI, HLI, RRP Mod 3: THI, ETI, ESI, RRP Mod 4: THI, ETI, ESI, HLI, HLI _New_	190.90 186.77 186.39 187.40	4.51 0.38 0.00 1.01	Mod 2 Mod 3	0.496 0.494	<0.001 <0.001
Fat	Mod 1: THI, ESI Mod 2: THI, ESI, RRP Mod 3: THI, ETI, ESI, RRP	224.53 226.79 228.94	0.00 2.26 4.41	Mod 1	0.1249	<0.01
Protein	Mod 1: THI, HLI _New_ Mod 2: THI, ETI, HLI _New_ Mod 3: THI, ESI, ETI, HLI _New_	225.50 227.68 229.74	0.00 2.18 4.24	Mod 1	0.1144	<0.01

**Table 6:** Results of LASSO and AIC analysis for finding the best multiple regression model between milk parameters and climatic indices in summer.

Layout Parameter	Preselected Predectors by LASSO	AIC	Δ AIC	Model Selected by AIC	*R*^2^	*p*-Value
Milk yield	Mod 1: THI , ETI, HLI Mod 2: THI, ETI, ESI Mod 3: THI, ETI, ESI, HLI	152.40 142.70 142.54	9.86 0.16 0.00	Mod 2 Mod 3	0.697 0.707	<0.001 <0.001
Fat	Mod 1: THI, ETI Mod 2: THI, ETI, HLI _New_ Mod 3: THI, ETI, ESI, HLI _New_ Mod 4: THI, ETI, ESI, HLI, HLI _New_ Mod 5: THI, ETI, ESI, HLI _New_, RRP	234.49 235.98 237.95 240.14 240.34	0.00 1.49 3.46 5.65 5.85	Mod 1	0.0103	<0.1
Protein	Mod 1: HLI, HLI _New_ Mod 2: ESI, HLI, HLI _New_ Mod 3: ESI, HLI, HLI _New_, RRP	231.82 229.92 225.36	6.45 4.56 0.00	Mod 3	0.1428	<0.01
